# Detecting Audio Adversarial Examples in Automatic Speech Recognition Systems Using Decision Boundary Patterns

**DOI:** 10.3390/jimaging8120324

**Published:** 2022-12-09

**Authors:** Wei Zong, Yang-Wai Chow, Willy Susilo, Jongkil Kim, Ngoc Thuy Le

**Affiliations:** 1 Institute of Cybersecurity and Cryptology, School of Computing and Information Technology, University of Wollongong, Wollongong, NSW 2522, Australia; 2 Department of Cyber Security, Ewha Womans University, Seoul 03760, Republic of Korea

**Keywords:** adversarial examples, automatic speech recognition, visualization, adversarial machine learning, adversarial example detection

## Abstract

Automatic Speech Recognition (ASR) systems are ubiquitous in various commercial applications. These systems typically rely on machine learning techniques for transcribing voice commands into text for further processing. Despite their success in many applications, audio Adversarial Examples (AEs) have emerged as a major security threat to ASR systems. This is because audio AEs are able to fool ASR models into producing incorrect results. While researchers have investigated methods for defending against audio AEs, the intrinsic properties of AEs and benign audio are not well studied. The work in this paper shows that the machine learning decision boundary patterns around audio AEs and benign audio are fundamentally different. Using dimensionality-reduction techniques, this work shows that these different patterns can be visually distinguished in two-dimensional (2D) space. This in turn allows for the detection of audio AEs using anomal- detection methods.

## 1. Introduction

Automatic Speech Recognition (ASR) systems are commonly used in many commercial applications. These systems are typically used to transcribe user speech into text and for recognizing user voice commands. Modern ASR systems rely on deep learning techniques, which have been shown to achieve better speech recognition performance in comparison with other traditional techniques [1,2,3]. Nevertheless, despite its success, deep learning techniques suffer from a variety of security threats [4]. Among these, Adversarial Examples (AEs) have emerged as a security threat that has attracted great interest within the research community.

Research on AEs first appeared in the image-recognition field, where small perturbations were applied to a benign (normal) image to generate an AE [5]. The goal of an AE is to fool deep learning models into classifying it under a different label while being perceived to be visually indistinguishable from the original image by a human. Research interest in AEs has rapidly spread to other areas, such as Natural Language Processing (NLP) [6,7,8], speech recognition [9,10,11,12,13,14], and speaker verification [15,16,17].

In conjunction with research on generating AEs, others have attempted to understand and explain the reasons for AEs. For example, Tsipras et al. [18] provided a provable demonstration, which showed that non-robust features referred to features that were weakly correlated with the corresponding label. They reasoned that AEs exist because classification is affected by such non-robust features in a data set. Ilyas et al. [19] affirmed the intrinsic existence of non-robust features in data sets. Others have also shown that perturbations in AEs can dominate classification. This can be seen as complementary to the presence of non-robust features in a data set [20].

Other researchers have focused on methods for defending against AEs. For instance, using intrinsic properties to differentiate between benign samples and AEs [21,22] and training of a classifier with both benign samples and AEs to detect previously unknown attacks [23,24]. Despite the fact that adversarial training was shown to be one of the most effective methods for defending against AEs [25], Zhang et al. [26] showed that successful AEs with small perturbations can still be generated if data points are far from the training set manifold.

While much research on AEs is in the image recognition field, this paper focuses on AEs in the audio domain. In the audio domain, while researchers have proposed methods for detecting audio AEs [27,28], the fundamental differences between audio AEs and benign audio are not well studied or understood. To date, there is a lack of research on visually analyzing audio AEs in relation to ASR systems. This paper investigates this by presenting a method of visually analyzing the intrinsic properties of audio AEs. We show that these properties can be used to differentiate audio AEs from benign audio. Consequently, this research demonstrates that it is possible to detect previously unknown audio AEs using these distinguishable features.

Our Contributions: This paper is an extended version of our work in [29]. We demonstrate that the decision boundaries around audio AEs are fundamentally different from the decision boundaries around benign audio. In our proposed method, we use heat maps to visualize the decision boundaries of ASR models in relation to changes in loss function values and normalized edit distances (Levenshtein distance). Using this approach, we show that both targeted and untargeted audio AEs have different decision boundary patterns in comparison with benign audio. By extracting features from decision boundaries and projecting them into two-dimensional (2D) space, this paper illustrates that targeted and untargeted AEs can clearly be separated from benign audio. As a result of this, we demonstrate the feasibility of using simple anomaly detection models to distinguish between AEs and benign audio.

The rest of this paper is organized as follows. Section 2 discusses related work that provides a background to our work. Our proposed method is described in Section 3. Section 4 discusses methods of generating attacks for detection, which is followed by the results of our experiments in Section 5. Section 6 discusses the robustness of AEs against our method, along with a promising direction for eliminating AEs. Finally, our conclusions and future work are presented in Section 7.

## 2. Related Work

In this section, we provide a discussion on related work, which provides a background to our work. This section starts by describing audio AEs. This is followed by the work on defending against such AEs. Then, it presents current techniques for visually analyzing AEs.

### 2.1. Audio Adversarial Examples

AEs were first presented in the image-recognition field [5] and can be categorized into targeted or untargeted AEs [30]. The difference between targeted and untargeted is that targeted AEs fool a model into outputting a predetermined result, whereas untargeted AEs simply cause a model to output an incorrect result. AEs can be generated under a white-box or black-box threat model. A white-box threat model assumes that an adversary knows everything about the target model. This includes its training data set, hyper-parameters, model weights, and so on. On the other hand, under a black-box threat model, an adversary is only able to obtain input and output pairs consisting of the AE and its corresponding result. Thus, black-box AEs are a subset of white-box AEs [31]. Interest in AEs has spread from the image domain to ASR systems. A summary of research efforts on audio AEs is presented in Table 1.

In early work on white-box audio AE generation by Yuan et al. [32], they hid malicious voice commands in songs to attack the Kaldi speech-recognition toolkit [33]. This work also demonstrated the transferability of the techniques whereby the generated AEs could be transferred to attack iFLYREC (https://www.iflyrec.com/, accessed on 1 November 2020). Transferability refers to the feature where an AE generated using one model is able to fool other models. Kaldi is a hybrid ASR model, i.e., Deep Neural Network–Hidden Markov Model (DNN-HMM), that outperformed traditional end-to-end DNN models. Carlini and Wagner [9] proposed a white-box method of generating audio AEs against DeepSpeech by optimizing the Connectionist Temporal Classification (CTC) loss. CTC loss is used for training sequence-to-sequence neural networks with unknown alignment between input and output sequences [34]. A limitation of their approach is that the max-norm of perturbations is used to reduce noise in the resulting audio AEs. Liu et al. [35] improved the quality and generation speed of the AE generation method proposed in [9] by partially optimizing perturbations. Other studies [10,36] have shown that there are better ways to suppress noise by incorporating psychoacoustics into the generation process. However, a study by Eisenhofer et al. [37] showed that a defender can deliberately remove inaudible components from input audio to avoid imperceptible adversarial perturbations. Furthermore, recent work in Zong et al. [38] demonstrated that high-quality audio AEs can be obtained without psychoacoustics.

**Table 1:** A summary of research efforts on audio AEs in ASR systems.

AE Type	Assumption	Method	Target Model
Targeted	White-box	Yuan et al. [32]	Kaldi
		Carlini and Wagner [9]	DeepSpeech
		Liu el al. [35]	DeepSpeech
		Schoenherr et al. [10]	Kaldi
		Qin et al. [36]	Lingvo [3]
		Zong el al. [38]	DeepSpeech
	Black-box	Taori et al. [39]	DeepSpeech
		Chen et al. [40]	Commercial products *
Untargeted	white-box	Neekhara et al. [41]	DeepSpeech
	Black-box	Abdullah et al. [42]	7 models ^#^

* including Google Assistant, Google Home, Microsoft Cortana, Amazon Echo, and IBM Speech to Text. ^#^ including Google (Normal), Google (Phone), Facebook Wit, DeepSpeech, DeepSpeech2, CMU Sphinx, and Microsoft Azure.

The generation of black-box audio AEs is more challenging compared with their white-box counterparts because the internal workings of the ASR model are not accessible to the adversary. Alzantot et al. [11] were the first to use genetic algorithms to generate black-box audio AEs. However, the target model in their study was a lightweight keyword spotting model, rather than an ASR model. In later work, Taori et al. [39] proposed black-box audio AEs against DeepSpeech. In addition to genetic algorithms, they also employed a gradient estimation technique to fine-tune perturbations when the edit distance between the transcribed and target phrases was small. Target phrases in their work were limited to two words. Chen et al. [40] proposed training a local surrogate model in which the decision boundaries resembled a target model. Audio AEs were generated using the surrogate model to attack a remotely deployed target. They demonstrated the success of their method in forcing commercial ASR products to output predefined commands.

Unlike targeted AEs, which output predefined commands, untargeted AEs are less malicious because they only cause a target model to output incorrect commands. Hence, untargeted audio AEs have received less attention in the research community. Under a white-box assumption, Neekhara et al. [41] proposed Universal Adversarial Perturbations (UAPs) that can be applied to any input audio to cause incorrect output. They empirically showed that their UAPs were more effective than random noise. Other work by Abdullah et al. [42] assumed black-box access to a target model. In particular, they proposed first decomposing input audio and then iteratively optimizing a threshold to eliminate components. The end goal was to identify an optimal threshold that preserves the perceptual quality of input audio while making a target model output incorrect results. They empirically showed that their method was able to cause commercial products to incorrectly transcribe input audio.

### 2.2. Defending against Audio Adversarial Examples

As audio AEs are a serious threat to the security of ASR systems, the past few years have witnessed an increase in the amount of research on defending against audio AEs. In this subsection, we present an overview of such techniques. It should be mentioned that the defense for other audio tasks, such as environmental sound classification [43], is beyond the scope of this paper.

There are two lines of work for defending against audio AEs, which are summarized in Table 2. The first line of work focuses on detecting the existence of adversarial perturbations in input audio. Zeng et al. [27] proposed the use of multiple ASR models to transcribe an input audio signal. If the resulting transcripts of these models diverged significantly, the audio would be classified as an AE. Their detection method is based on the assumption that audio AEs cannot be transferred between multiple ASR models. On the other hand, this method requires the deployment of multiple ASR systems, which may not be practical in real-world applications. Another defense method, proposed by Yang et al. [28], detects audio AEs based on temporal dependency. They observed that unlike benign audio, audio AEs cannot preserve temporal dependency. Specifically, they observed that the transcripts of partial audio AEs, e.g., the first half of an AE, were significantly different compared with the transcripts of the full AEs. In contrast, there was significant overlapping between the transcripts of partial and full input for benign audio.

Recently, Samizade et al. [44] proposed a defense method where they trained a Convolutional Neural Network (CNN) on the spectrograms of benign audio and AEs and demonstrated that it could detect audio AEs with high accuracy. Nonetheless, their method suffered from low performance when detecting previously unknown attacks. This is due to the lack of generalization in out-of-distribution data. Improving the performance for out-of-distribution data is still an active topic in the deep learning literature [49]. Guo et al. [45] proposed another audio AE detection method, which was based on an efficient multivariant partition based method for extracting features. Although their method demonstrated high performance in detecting audio AEs, the requirement of training a classifier on both attacks and clean audio may make it impractical when attacks are unknown in advance. Another recent study by Hussain et al. [46] proposed a framework for detecting audio AEs. The underlying idea behind their method is that transcripts of audio AEs will be significantly altered if small modifications to the audio are introduced, whereas transcripts of clean audio input will be stable. They thus proposed applying transformations to input audio, and an audio AE was detected if its transcript changed significantly. Although the methods discussed above have successfully detected audio AEs, the intrinsic properties that differentiate audio AEs from benign audio are not well studied or understood.

The other line of work in this area focuses on recovering the original clean audio by destroying adversarial perturbations in audio AEs. Yang et al. [47] used a speech-quality-enhancement network to preprocess audio AEs. After adversarial training, this network can efficiently remove adversarial perturbations. However, adversarial training introduces a tradeoff between model performance and robustness [50], which may limit the performance of the speech-quality-enhancement network. Guo et al. [48] observed that denoising techniques resulted in different performances after removing adversarial perturbations in different audio AEs. Hence, they proposed an intelligent noise-reduction method, called INOR, which was effective in removing adversarial perturbations from different audio AEs. Yuan et al. [32] and Chen et al. [40] observed that audio downsampling was able to destroy adversarial perturbations. Nonetheless, they performed experiments on their proposed audio AEs.

### 2.3. Visualization Techniques for Analyzing Adversarial Examples

Visualization is useful for facilitating the understanding of deep learning techniques [51]. Several visualization efforts have focused on aiding in the intuitive understanding of AE properties. In an initial attempt, Norton et al. [52] built a web-based interface to interactively show the image AE generation process. Seminal work conducted by Liu et al. [53] visually explained the transferability of image AEs. In their work, they visualized the decision boundaries of several image recognition models and found that AEs could be transferred due to their overlapping decision boundaries. In other work, Stutz et al. [54] showed that image AE perturbations are interpretable if the AEs are constrained on the manifold of a data set. In addition, different patterns in the loss function gradients of input images in non-robust and robust models have been visually compared [18]. A study by Zhang et al. [20] visualized logit vectors of a model in relation to an image AE, along with its corresponding clean image and perturbations. The experimental results in their study showed that logit vectors of an image AE and their corresponding perturbations are correlated.

Despite these research efforts on visualization for AEs, to date, there is limited research on the visual analysis of audio AEs in the ASR domain. This paper fills this gap by proposing a method of visually analyzing the intrinsic properties that can be used to distinguish audio AEs from benign audio.

## 3. Proposed Method

The research in this paper proposes a method of visually analyzing targeted and untargeted audio AEs. This section presents the details of our proposed method.

### 3.1. Visualizing Decision Boundaries

In general, benign audio is significantly more robust than audio AEs. Robustness refers to whether audio can be transcribed correctly despite the presence of noise. Benign audio is generally more robust as it can usually be transcribed correctly even when additional noise is added to the audio signal. This implies that the decision boundary patterns around benign audio are potentially different from those of audio AEs. Hence, we propose a method of visualizing the decision boundaries of ASR models to show this difference.

Unlike in image recognition, where there are usually fixed sets of labels, the decision boundaries of ASR models are more difficult to visualize as an audio signal can potentially be transcribed into a large number of output strings. Moreover, if one were to simply treat different transcripts as different labels, the visualization results would be confusing. This is because a difference between labels cannot appropriately represent the difference in the transcribed text. For example, if “paper” and “papers” were to be treated as two different labels, such as 1 and 2 in numeric form, information on the similarity between these two transcripts will be lost. Therefore, it makes more sense to visualize the decision boundaries of an ASR model via changes in the resulting transcripts when input audio is modified. Moreover, changes in the loss function values can also be used to represent the decision boundary patterns of an ASR model.

In this paper, we propose a method of visualizing the decision boundaries of ASR models using heat maps to show changes in loss function values and changes in normalized edit distances. The reason for using heat maps is that they can clearly represent changes in values visually. The proposed method is formally defined here. Let *x* be the input audio and *y* be the transcript of *x*, which may be different from the ground truth if the audio signal is incorrectly transcribed; let f() be the ASR model; and let $\ell_{net}\left(\right)$ be the corresponding loss function. We can calculate the gradient of the loss function with respect to *x* as $\vec{g}=\nabla \ell_{net}\left(f\left(x\right),y\right)$ and normalize it to be of unit length using $\bar{g}=\frac{\vec{g}}{\left|\right|\vec{g}{\left|\right|}_{2}}$. Then, we initialize a random unit vector $\bar{q}$ that is not parallel to $\bar{g}$. We obtain $\vec{p}=\bar{q}-\left(\bar{q}\cdot \bar{g}\right)\times \bar{g}$ and $\bar{p}=\frac{\vec{p}}{\left|\right|\vec{p}{\left|\right|}_{2}}$. Thus, $\bar{p}$ is of unit length and perpendicular to $\bar{g}$.

The heat maps of loss function values and normalized edit distances are defined as the square matrices $M_{loss}$ and $M_{edit}$, respectively. The size of both matrices is n×n. Let *s* be a predefined number controlling the extent to which *x* is modified. The definition of $M_{loss}$ and $M_{edit}$ is shown in Equation (1).

(1) $$\begin{array}{cc} \; & {\left[M_{loss}\right]}_{i,j}=\ell_{net}\left(f\left(x+s_{i}\cdot \bar{p}+s_{j}\cdot \bar{g}\right),y\right)\\ \; & {\left[M_{edit}\right]}_{i,j}=\frac{d_{edit}\left(f\left(x+s_{i}\cdot \bar{p}+s_{j}\cdot \bar{g}\right),y\right)}{h_{length}\left(y\right)} \end{array}$$

where $s_{k}=\frac{-n\cdot s}{2}+\frac{n\cdot s\cdot (k-1)}{\left(n-1\right)}$, $d_{edit}\left(\right)$ is the function to calculate the edit distance and $h_{length}\left(\right)$ returns the transcript length, which is used to normalize the edit distance. In Equation (1), the audio data are evenly modified along $\bar{g}$ and $\bar{p}$ via a step size *s*. Normalizing the edit distance is necessary because edit distance by itself cannot fairly compare the change in transcripts for different *y*. For example, a small edit distance means a more severe change for short *y* than for long *y*.

### 3.2. Feature Extraction

For additional insight into the heat maps, two-dimensional (2D) reduction techniques were used to project the results into 2D space to identify potential patterns. For this, we used the Principal Component Analysis (PCA) [55] and the t-distributed Stochastic Neighbor Embedding (t-SNE) [56] techniques. A simple feature extraction method was employed in this study. While more advanced methods, such as training a convolutional neural network on heat maps, may potentially produce better results, a simple method serves as a low bound that can be improved on. An investigation into feature extraction methods for the proposed method is a potential direction for future work.

Given input audio, we calculate a vector $v_{loss}$ based on the change in loss function values if we modify the audio along the gradient direction $\bar{g}$ and a perpendicular direction $\bar{p}$. $\bar{g}$ and $\bar{p}$ were previously defined in Section 3.1. Similarly, we calculate a vector $v_{edit}$ based on the change in normalized edit distances. As defined in Equation (2), the feature vector $v_{ft}$ representing the heat maps of an input audio is simply a concatenation of $v_{loss}$ and $v_{edit}$. In other words, $v_{ft}$ measures both the change in loss function values and normalized edit distances when input audio is modified. Intuitively, using $v_{ft}$ would result in better performance in distinguishing audio AEs from benign audio than using only $v_{loss}$ or $v_{edit}$. For simplicity, we refer to $v_{ft}$ as the features of input audio.

(2) $$\begin{array}{cc} v_{loss} & =\left[\begin{array}{c} \ell_{net}\left(f\left(x+\bar{g}\right),y\right)-\ell_{net}\left(f\left(x\right),y\right)\\ \ell_{net}\left(f\left(x-\bar{g}\right),y\right)-\ell_{net}\left(f\left(x\right),y\right)\\ \ell_{net}\left(f\left(x+\bar{p}\right),y\right)-\ell_{net}\left(f\left(x\right),y\right)\\ \ell_{net}\left(f\left(x-\bar{p}\right),y\right)-\ell_{net}\left(f\left(x\right),y\right) \end{array}\right]\\ v_{edit} & =\left[\begin{array}{c} d_{edit}\left(f\left(x+\bar{g}\right),y\right)/h_{length}\left(y\right)\\ d_{edit}\left(f\left(x-\bar{g}\right),y\right)/h_{length}\left(y\right)\\ d_{edit}\left(f\left(x+\bar{p}\right),y\right)/h_{length}\left(y\right)\\ d_{edit}\left(f\left(x-\bar{p}\right),y\right)/h_{length}\left(y\right) \end{array}\right]\\ v_{ft} & =\left[\begin{array}{c} v_{loss}\\ v_{edit} \end{array}\right] \end{array}$$

## 4. Attack Generation

This section discusses the method of generating AEs for detection.

### 4.1. Targeted Audio Adversarial Examples

This paper analyzes an improved version of the state-of-the-art targeted audio AE generation process proposed by Carlini and Wagner [9]. In their research, distortion caused by perturbations was measured by comparing the level of perturbations δ, in decibels (dB), with the original waveform *x*. The calculation is given as $dB_{x}\left(\delta \right)=dB\left(\delta \right)-dB\left(x\right)$, where $dB\left(x\right)=\max_{i}20\cdot log_{10}\left(x_{i}\right)$, which is used in the formulation shown in Equation (3) [9].

(3) $$\begin{array}{cc} \; & {minimize\;||\delta ||}_{2}^{2}+c\cdot \ell_{net}\left(f\left(x+\delta \right),y\right)\\ \; & such\;that\;dB_{x}\left(\delta \right)\leq \tau \;\;\; \end{array}$$

where τ limits the max-norm of δ, ${\left||\delta |\right|}_{2}^{2}$ is the squared Euclidean norm of δ, f() represents the ASR model, *y* is the target phrase, $\ell_{net}\left(\right)$ represents the loss function of the ASR model, and *c* is used as a trade-off between the amount of adversarial perturbation and making δ small.

A major drawback of this method is that perturbations are limited by the max-norm, which is arguably not suitable for minimizing noise in audio AEs. This is because max-norm constrained perturbations are applied in a non-selective manner, such that noise is clearly audible during quiet sections. In contrast, Qin et al. [36] showed that it is more appropriate to incorporate psychoacoustics to suppress noise in audio AEs. Using their approach, they divide the generation process into two stages. In the first stage, a targeted audio AE is generated in the same way as [9]. Then, the second stage tries to limit perturbations to be under the masking threshold that was proposed in [57]. The formulation to solve this is shown in Equation (4) [36], where $l_{\theta }\left(\right)$ is the loss function to calculate the hinge loss of the masking threshold and α controls the trade-off between the amount of adversarial perturbation and it being imperceptible.

(4) $$\begin{array}{cc} \; & minimize\;\ell_{net}\left(f\left(x+\delta \right),y\right)+\alpha \cdot l_{\theta }\left(x,\delta \right)\;\;\; \end{array}$$

It should be noted that limiting the max-norm of perturbations in stage 1 is somewhat unnecessary since the original purpose of limiting the max-norm is to suppress noise. Furthermore, in their approach, noise suppression is also done in stage 2.

Based on the method in Qin et al. [36], we improved the targeted AEs generation process proposed by Carlini and Wagner [9] by constraining perturbations via the masking threshold instead of the max-norm. Specifically, we solve the formula in Equation (5), where *X* represents the set of valid audio data, ${\left||\delta |\right|}_{2}^{2}$ is the squared Euclidean norm of δ, $\ell_{net}$ is the loss function of the ASR model, $l_{\theta }$ is the hinge loss of the masking threshold from [36], and β and α are factors used to balance the different losses. There are still two stages. During stage 1, a targeted audio AEs is generated with α set to 0, so that the hinge loss of the masking threshold will have no contribution. During stage 2, α is set to a small value, e.g., 0.05, to suppress noise.

As asserted in [9], limiting the max-norm of perturbations would often result in the optimization not converging, but rather oscillating around a solution. In contrast, we do not limit the max-norm of perturbations in Equation (5), thereby potentially reducing AE generation time.

(5) $$\begin{array}{cc} \; & {minimize\;||\delta ||}_{2}^{2}+\beta \cdot \ell_{net}\left(f\left(x+\delta \right),y\right)+\alpha \cdot l_{\theta }\left(x,\delta \right)\\ \; & such\;that\;x+\delta \in X \end{array}$$

### 4.2. Untargeted Audio Adversarial Examples

To the best of our knowledge, to date, there is limited research on untargeted audio AEs. This is because untargeted audio AEs are less interesting compared to targeted AEs, since they only lead to wrong or even meaningless transcripts. Nevertheless, for the sake of completeness, we also analyze untargeted audio AEs in this research.

We devised two approaches to generate untargeted audio AEs. The first is based on the Fast Gradient Sign Method (FGSM) [25]. This method simply takes one step along the gradient of the loss function with respect to the input audio. Perturbations δ are calculated as in Equation (6) [25], where *x* is the input audio, *y* is the target phrase, $\ell_{net}\left(\right)$ is the loss function, and ϵ is the step size.

(6) $$\begin{array}{cc} \; & \delta =\epsilon \cdot sign(\nabla_{x}\ell_{net}\left(f\left(x\right),y\right))\;\;\; \end{array}$$

An audio AE $x^{\prime }$ is then calculated as: $x^{\prime }\leftarrow x-\delta$. While this will not generate targeted audio AEs, like the method in [9], this method can generate untargeted audio AEs if we set *y* to be the reversed ground truth. The reversed ground truth is typically different from the original. An untargeted AE is successfully generated if the edit distance between the transcript and the ground truth exceeds a certain threshold. Edit distance is defined as the minimum number of letter-level modifications, including insertions, deletions, and substitutions, required to change one text to another.

The second approach to generating untargeted audio AEs was inspired by the black-box targeted audio AE proposed by Taori et al. [39], where they used a genetic algorithm to search for perturbations that led to an ASR outputting a target phrase. When the transcript of the best solution is within a predefined edit distance of the target phrase, the generation process uses a gradient estimation strategy to continue the search process. In this work, we use the gradient estimation strategy in [39] to generate untargeted audio AEs. We also incorporate the noise suppression technique from [36] in the generation process. As shown in Algorithm 1, we first reverse the ground truth and use the reversed transcript as the target for optimizing the input audio, as in the first approach of generating untargeted audio AEs via FGSM. The generation is deemed to be successful when the edit distance between the transcript and the ground truth exceeds a certain threshold.  
**Algorithm 1** Untargeted Audio AE Generation**Input:** original audio signal, *x*; ground truth transcript, *y*; target ASR model *m*; maximum iteration: max_iter; edit distance threshold: distance_min**Output:** black-box untargeted audio AE, $x^{\prime }$$x^{\prime }$←*x*y_reverse← reverse the characters in *y*While iter<max_iter do  y_reverse_loss← calculate loss of y_reverse  grad_estimate← estimate the gradient of the loss function $x^{\prime }$ using y_reverse_loss  $x^{\prime }$←$x^{\prime }$-grad_estimate * learning_rate  // use the lowering noise technique from [36]  masking_loss← masking loss noise in $x^{\prime }$  optimize masking_loss noise in $x^{\prime }$  If EditDistance(*y*, transcript of $x^{\prime }$) ≥distance_min   return $x^{\prime }$  End IfEnd WhileIf iter==max_iter  return failEnd If

## 5. Experiments and Discussion

### 5.1. Target Models and Data Sets

As with similar research in the ASR domain [9,10,39,44], we used DeepSpeech [1] as one of the target models for our experiments. DeepSpeech 0.8.2 (DeepSpeech 0.8.2 was implemented by Mozilla https://github.com/mozilla/DeepSpeech (accessed on 1 November 2020)), which is the latest release at the time of writing, was used in this research. It should be noted that DeepSpeech 0.1, which was used in previous studies [9,39], has been superseded with newer versions. In addition, DeepSpeech2 [2], which is an improved version of DeepSpeech that employs an end-to-end architecture, was also used. We used DeepSpeech2 V2 (DeepSpeech2 V2 was implemented and released by Sean Naren https://github.com/SeanNaren/deepspeech.pytorch (accessed on 1 November 2020)).

LibriSpeech [58] was employed as the data set because DeepSpeech and DeepSpeech2 both provide pre-trained models on LibriSpeech. In the experiments, we used audio from the *test-clean* and *dev-clean* data sets. For targeted AEs, one of the target phrases “power off”, “turn on airplane mode”, “visit danger dot com”, “call malicious number”, and “turn off lights” was selected at random to mimic malicious voice commands. The generation of untargeted AEs was deemed to be successful if the edit distance between the transcript and the ground truth was larger than 40% of the ground truth.

In previous work by Carlini and Wagner [9], they generated audio AEs using the first 100 test instances of the Mozilla Common Voice data set [59]. Most of this audio was short, between 1 and 8 seconds in duration. Carlini and Wagner [9] empirically observed that the generation of targeted AEs was easier the longer the source phrase, while the generation would be more difficult the longer the target phrase. Since our target phrases were relatively short, we used audio below 5 seconds to balance the difficulty of generating targeted audio AEs.

All experiments in this paper were performed with an Intel i7-8750H CPU and an Nvidia GeForce GTX 1060 graphic card. Using randomly selected audio from the *test-clean* data set of DeepSpeech and DeepSpeech2, respectively, we generated 150 targeted AEs, 150 untargeted AEs using FGSM, and 150 untargeted AEs based on our proposed method. For simplicity, in the remainder of this paper, we refer to untargeted AEs using our proposed method as untargeted AEs and untargeted AEs using FGSM as FGSM AEs. To obtain a balanced data set, we also extract 150 correctly transcribed and 150 incorrectly transcribed audio from the *test-clean* data set of each model. In addition, we generated 150 noisy audio signals by applying Gaussian noise with a standard deviation of 0.01.

To generate targeted AEs, we ran 350 epochs for DeepSpeech and 300 epochs for DeepSpeech2 to suppress noise during the second stage since we observed that it is easier for DeepSpeech2 to suppress the noise without destroying adversarial perturbations. Noise suppression in all targeted AEs against DeepSpeech2 was successful. However, some AEs against DeepSpeech failed to lower noise within the 350 epochs. As such, we individually fine-tuned these noisy AEs by running extra epochs until the masking loss ($l_{\theta }\left(\right)$ in Equation (5)) was below a specific threshold. The smaller the masking loss, the smaller the distortion perturbations caused. We set the threshold to the masking loss calculated using the −20 dB distortion set published by [9] (https://nicholas.carlini.com/code/audio_adversarial_examples (accessed on 1 November 2020)).

**Figure 1:**
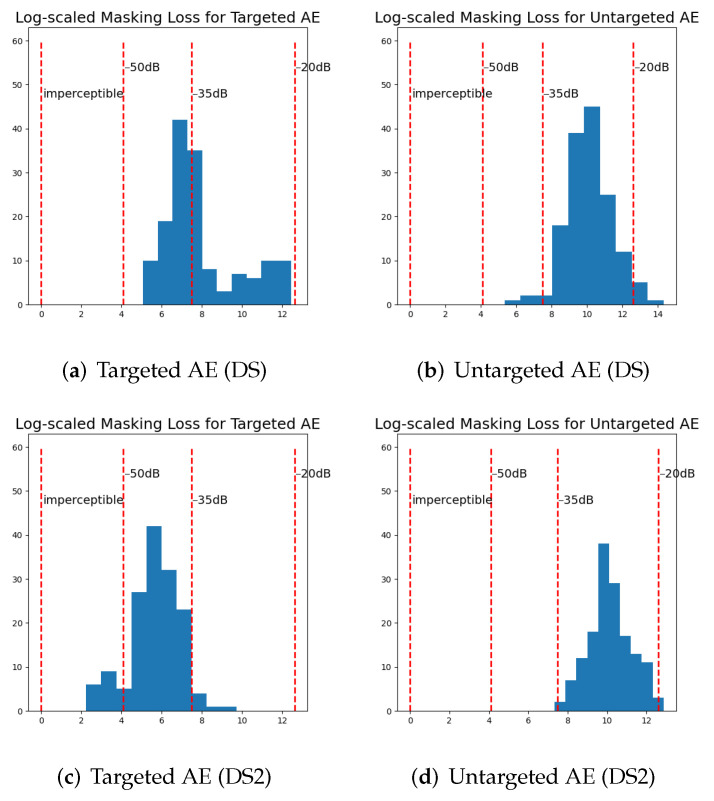
Histograms comparing the masking loss ($l_{\theta }\left(\right)$ in Equation (5)) of our generated adversarial examples (AEs) for DeepSpeech (DS) and DeepSpeech2 (DS2) with the −20 dB distortion, −35 dB distortion, and −50 dB distortion sets published by [9] (labeled as −20 dB, −35 dB, and −50 dB, respectively) and the first set of imperceptible adversarial examples published by [36] (labeled as imperceptible). The smaller the masking loss, the lower the resulting distortion perturbations. Coordinates along the horizontal axis were calculated as ln(maskingloss+1). Overall, the masking loss of targeted AEs against DeepSpeech2 was smaller compared with DeepSpeech. The masking loss of most untargeted AEs was between −20 dB and −35 dB.

**Figure 2:**
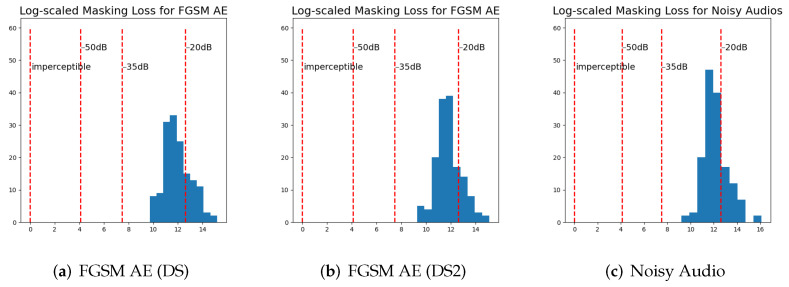
Histograms comparing the masking loss of fast gradient sign method (FGSM) AEs and noisy audio for DeepSpeech (DS) and DeepSpeech2 (DS2) with the −20 dB distortion, −35 dB distortion, and −50 dB distortion sets published by [9] (labeled as −20 dB, −35 dB, and −50 dB) and the first set of imperceptible adversarial examples published by [36] (labeled as imperceptible). Coordinates of the horizontal axis are calculated as ln(loss+1), where loss is the masking loss proposed by [36]. We can see that the masking loss of FGSM AEs is similar to the masking loss of noisy audio.

The masking losses of our AEs were compared with the −20 dB distortion, −35 dB distortion, and −50 dB distortion sets published by [9] and the first set of the imperceptible adversarial examples published by [36] (http://cseweb.ucsd.edu/~yaq007/imperceptible-robust-adv.html (accessed on 1 November 2020)). Figure 1 and Figure 2 show these results. Smaller dB values mean lower distortion. Carlini and Wagner [9] reported that the distortion of 95% of their targeted AEs ranging between −15 dB and −45 dB. Thus, the resulting distortion in our targeted AEs is comparable with the results of related work. It should be mentioned that we can further lose masking loss by running more epochs, which will require a longer generation time. We have made examples of AEs generated in this work available at https://drive.google.com/drive/folders/1Ffed7xHmP5oKCuypEgJxQ80p35-vSIBm?usp=sharing (accessed on 20 October 2022).

Table 3 shows a comparison of the time taken for generating the audio AEs. FGSM was the fastest approach, but it had the lowest success rate. On average, it took 2.4 and 7.0 min to generate targeted audio AEs for DeepSpeech and DeepSpeech2, respectively. On the other hand, our proposed method required an average of 4.4 and 4.9 min to generate untargeted audio AEs. While we generated AEs one at a time, the generation process can be accelerated by generating multiple AEs in parallel. As a loose comparison, Carlini and Wagner [9] reported that their approach took about one hour to generate a single targeted audio AE on commodity hardware, while Zeng et al. [27] reported a time of 18 min on an 18-core CPU with dual graphic cards. While we cannot conclude that our generation process is statistically faster as the source audio and target phrases were different, intuitively, our method should speed up the generation of AEs because we do not limit the max-norm of perturbations.

### 5.2. Visualizing Decision Boundaries

**Figure 3:**
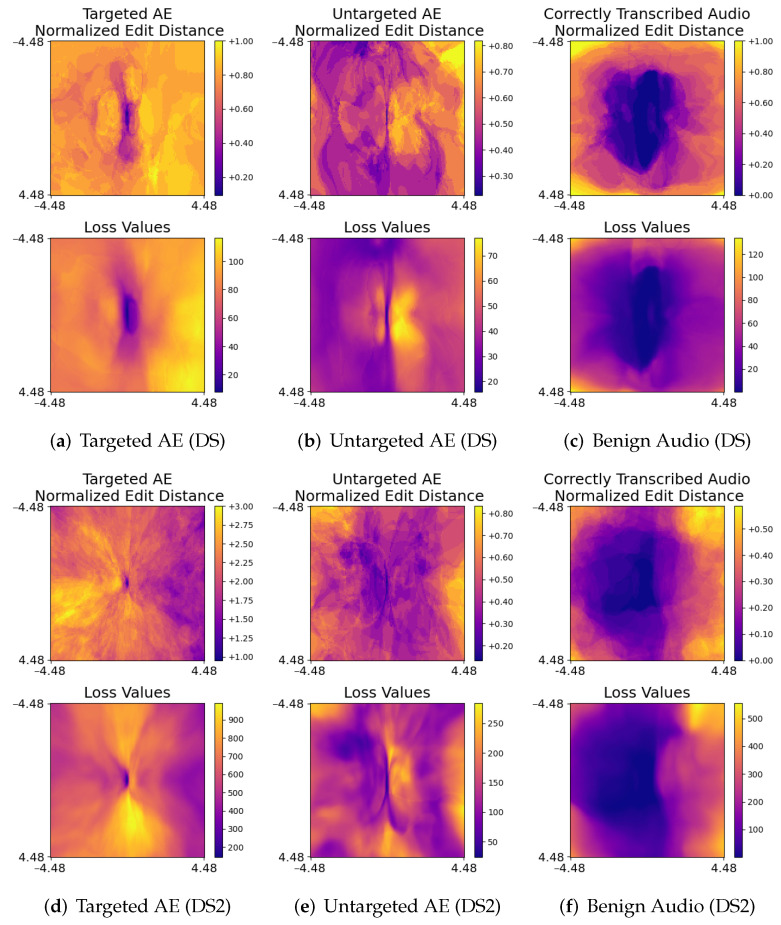
Heat maps of loss function values and normalized edit distances regarding different audio AEs and benign audio for DeepSpeech (DS) and DeepSpeech2 (DS2), respectively. Changes in loss function values and normalized edit distances of targeted and untargeted audio AEs are clearly different from benign audio.

As described in Section 3, the proposed method represents the decision boundaries of ASR models using heat maps of loss-function values and normalized edit distances. The $M_{loss}$ and $M_{edit}$ were calculated for correctly transcribed benign audio, targeted, and untargeted audio AEs. It was empirically observed that good results could be produced using a matrix of 128×128 and a step size *s* of 0.07. Figure 3 shows examples of resulting heat maps.

In the heat maps shown in Figure 3, the horizontal axis represents the direction of the gradient of the loss function the input audio, while the vertical axis represents a random direction that is perpendicular to the gradient. The heat maps were generated by modifying input audio along these two directions and recording the changes. The center of the heat maps represents unmodified audio. In the experiments, we set *y* in Equation (1) to the transcript of the unmodified audio, because we wanted to calculate the changes in loss values and transcripts when modifying audio. For example, *y* is set to the target phrase of a targeted audio AE or the incorrect transcript of an untargeted audio AE.

It is evident from the resulting patterns that changes in loss function values and normalized edit distances are correlated. This aligns with the intuition that loss function values returned by an ASR model should increase as the difference between the transcript and *y* increases and vice versa. Furthermore, we can see that when a targeted audio AE is modified slightly, the resulting loss function value and normalized edit distance change significantly. This is true for both DeepSpeech and DeepSpeech2 and is consistent with our observation that adversarial perturbations in the generated targeted audio AEs are not robust. The significant changes in loss function values and normalized edit distances when we modify AEs are an indication of the non-robust property of adversarial perturbations.

In contrast, changes in loss function values and normalized edit distances for correctly transcribed benign audio are significantly smaller than for targeted audio AEs when audio is slightly modified. This implies that correctly transcribed benign audio is much more robust against perturbations than targeted audio AEs. This is consistent with our observation that some correctly transcribed benign audio could still be correctly transcribed even when a large amount of noise is present. Another observation is that slightly modifying untargeted audio AEs also results in large changes in loss function values and normalized edit distances. However, while this change appears to be less severe than targeted audio AEs, the resulting patterns are different when compared with the results of correctly transcribed benign audio.

### 5.3. Dimensionality Reduction

Based on the different patterns in loss function values and normalized edit distances in relation to targeted and untargeted audio AEs and benign audio shown in Section 5.2, it is logical to consider the possibility of differentiating audio AEs from benign audio-based differences in their patterns. Thus, we extracted features from the audio and projected them into 2D space using the PCA and t-SNE methods, using the method described in Section 3. It should be noted that if audio AE and benign audio features can clearly be differentiated into 2D space, this indicates that they can also be separated in the original higher-dimensional space.

**Figure 4:**
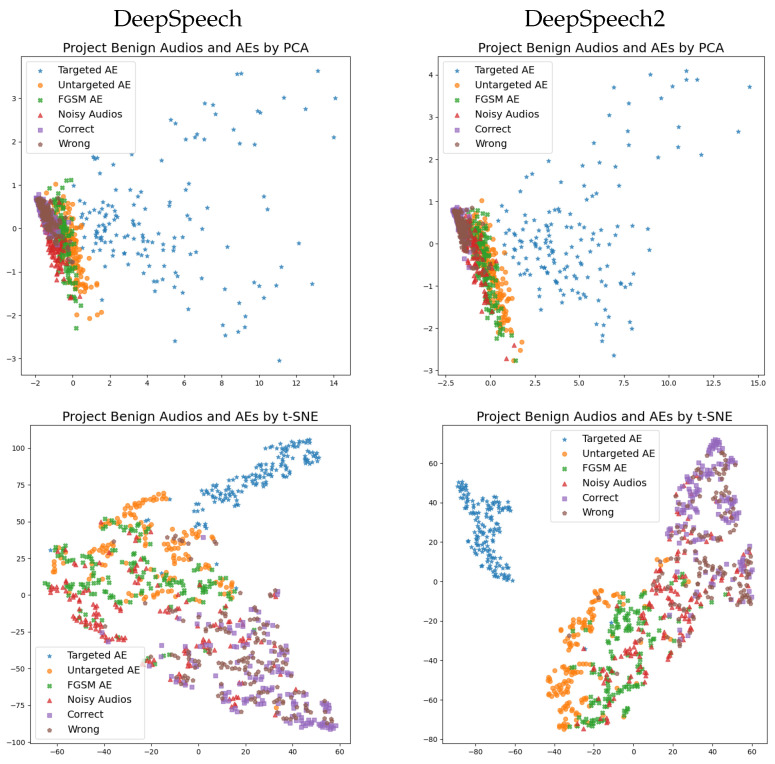
Results obtained by projecting the features of various types of audio using the principal component analysis (PCA) and t-distributed stochastic neighbor embedding (t-SNE) techniques.

In the experiment, benign audio was grouped as correctly and incorrectly transcribed audio. This was carried out to investigate whether there was a difference between them. In addition, noisy audio was also included. The features were normalized using their mean values and standard deviation before projecting them in 2D space. These results are shown in Figure 4.

The PCA projection results were almost the same for DeepSpeech and DeepSpeech2. Correctly and incorrectly transcribed audio clustered around the origin, while the other audio types were spread away from the origin. The correctly and incorrectly transcribed audio almost overlapped, indicating that there is little difference between their features. As previously discussed, the changes in loss function values and normalized edit distances for correctly transcribed benign audio are small, which explains why correctly and incorrectly transcribed audio cluster around the origin. In contrast, targeted audio AEs are far away from the origin. This is because small modifications will result in significant changes for targeted audio AEs, as discussed in the previous section. Untargeted audio AEs, FSGM audio AEs, and noisy audio all spread slightly away from the origin in the same direction. This implies that the features of these three audio types are similar.

Compared with PCA results, t-SNE projection was better at visualizing relationships between the data samples. In Figure 4, t-SNE projection again shows similar results for DeepSpeech and DeepSpeech2. Three clusters, excluding noisy audio, can be identified as follows: targeted audio AEs are clearly grouped in the first cluster; the second cluster mainly contains correctly and incorrectly transcribed benign audio; and the third cluster consists of untargeted audio AEs and FGSM AEs, i.e., untargeted attacks. The results of t-SNE projection are promising, since the various audio types are clustered according to their categories. An interesting observation is that incorrectly transcribed audio does not overlap with untargeted audio AEs or FGSM AEs, although all of them lead to incorrect transcriptions. A potential explanation is that incorrectly transcribed audio from the *test-clean* data set does not cause severe errors such as untargeted audio AEs and FGSM AEs. In addition, noisy audio is contained in both the second cluster (benign audio) and the third cluster (untargeted attack). This may be because some noisy audio is like benign audioin that it can be transcribed correctly or with little error, while some noisy audio behaves like untargeted attacks, which lead to significant errors in transcriptions. Upon closer inspection, the untargeted AEs and FGSM AEs are separate from each other in the case of DeepSpeech2, but the same is not true for DeepSpeech.

### 5.4. Anomaly Detection

Visualization results presented in the previous subsection indicate the possibility of detecting audio AEs based on their features. Hence, instead of training a classifier on benign audio and audio AEs, we experimented with using anomaly detection to detect audio AEs. In practice, audio AEs generated by adversaries are unlikely to be previously seen. Anomaly detection is appropriate for defending against previously unknown attacks.

In the experiments, we used audio from the *dev-clean* data set to train an anomaly detection model. This model was then used to detect audio AEs generated using the *test-clean* data set. In particular, audio features from *dev-clean* were extracted using the method described in Section 3. These features were used to train an EllipticEnvelope model implemented by scikit-learn [60]. This model detects outliers in a Gaussian distributed data set. We use the default parameters so that our experiment results can serve as a lower bound for anomaly detection. We report true positive (TP), false positive (FP), true negative (TN), false negative (FN), and detection rate (DR) for each category of benign audio and audio AEs together with overall precision (Pre), recall (Rec), and accuracy (Acc). Specifically, $precision=\frac{TP}{TP+FP}$, $recall=\frac{TP}{TP+FN}$, $accuracy=\frac{TP+TN}{TP+FP+TN+FN}$. For audio AEs, $DR=\frac{TP}{TP+FP}$. For benign audio, $DR=\frac{TN}{TN+FN}$.

Table 4 presents the experimental anomaly detection results for DeepSpeech and DeepSpeech2. Overall, the detection results are similar for both ASR models. As expected, targeted AEs are easily detected at detection rates of 100%. This is in line with the observation that targeted AEs can clearly be separated from other audio types in lower-dimensional space. It is reasonable that the detection rates of untargeted AEs were loan-targeted AEs since some untargeted AEs were mixed with benign audio in the PCA projection, as previously shown in Figure 4. The detection rates of FGSM AEs were surprisingly lone untargeted audio AEs, although these two AEs were clustered together in the t-SNE projection. This indicates that the simple anomaly detection model that was used is too basic for detecting FGSM AEs. In addition to benign audio, noisy audio could also be correctly identified at high detection rates. This was not as expected, since some of the noisy audio was mixed with untargeted AEs and FGSM AEs in low-dimensional space. This suggests that noisy audio is actually clustered with benign audio in the original higher-dimensional space, even though the 2D projection did not show this.

In a study by Samizade et al. [44], they generated white-box and black-box targeted audio AEs against DeepSpeech. They trained a neural network on white-box targeted audio AEs to detect black-box targeted audio AEs and vice versa. Our detection accuracy for the two ASR models of 87.44% and 82.22% is overall higher than their reported results of 82.07% and 48.76%, respectively. While this may not be a fair comparison, as they used a different approach, we mainly want to emphasize that the detection of previously unknown audio AEs is a challenging task. It is anticipated that if we extract more sophisticated features and utilize a more advanced anomaly detection method, it is highly likely that the detection results can be improved.

## 6. Discussion

In this section, we start by discussing the robustness of AEs against our method. Then, we discuss a promising research direction for eliminating AEs.

### 6.1. Robust Audio Adversarial Example

The fundamental assumption underlying this research is that the decision boundary patterns around benign audio and audio AEs are significantly different from one another. In this study, we used heat maps of loss function values and normalized edit distances to test the validity of this assumption. We also investigated whether the heat maps could differentiate audio AEs from benign audio under a white-box threat model. Although we demonstrated that heat maps of audio AEs and benign audio are significantly different, these audio AEs were generated without prior knowledge of the heat map generation process. It is conceivable that if an adversary has full knowledge of how the heat maps are generated, they can potentially generate targeted audio AEs with small changes in loss function values and normalized edit distances when the AEs are modified. We refer to this type of AEs as robust audio AEs because they are potentially indistinguishable from benign audio using our proposed heat map visualization method. Moreover, features extracted from such robust audio AEs may not be distinct from benign audio features.

In research efforts to increase the robustness of AEs, Athalye et al. [61] proposed the use of Expectation over Transformation (EoT). The idea behind this approach is to optimize the loss function over various transformations, such as Gaussian noise. Qin et al. [36] employed this method to incorporate reverberations in the generation process in order for audio AEs to remain adversarial over the air. If such reverberations were used to generate audio AEs, it is possible that there may be fewer changes in loss function values and normalized edit distances for such robust AEs, at least in the directions considered to be transformations. This is because the EoT directly incorporates this property in the optimization formula. From another point of view, the EoT can be thought of as imposing limits on the resulting decision boundary patterns around successfully generated AEs.

**Figure 5:**
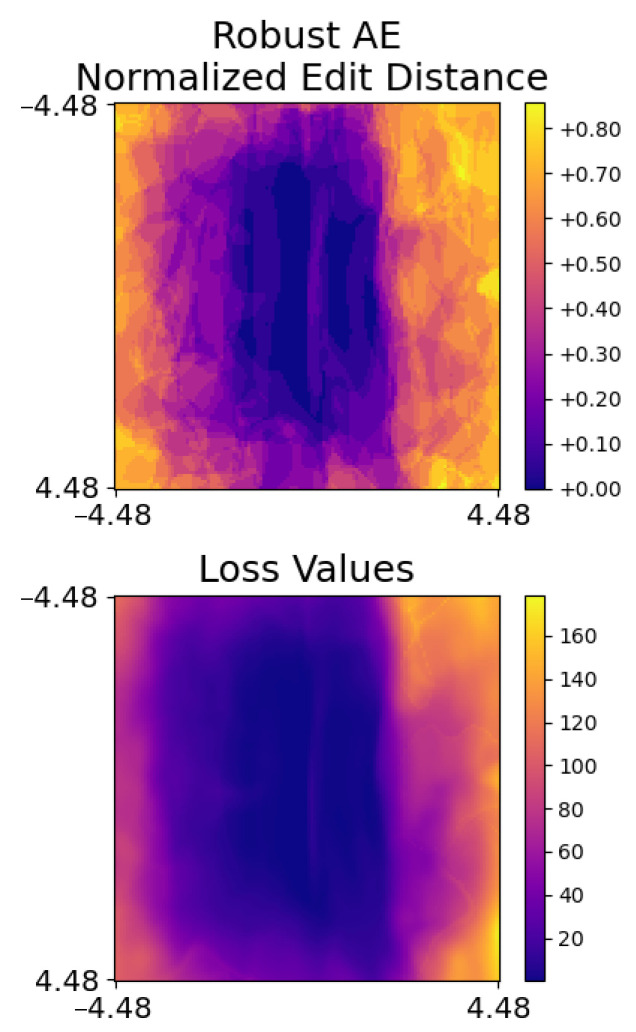
Heat maps of loss function values and normalized edit distances for a robust audio AE. There are small changes in loss function values and normalized edit distances when this robust audio AE is slightly modified.

**Figure 6:**
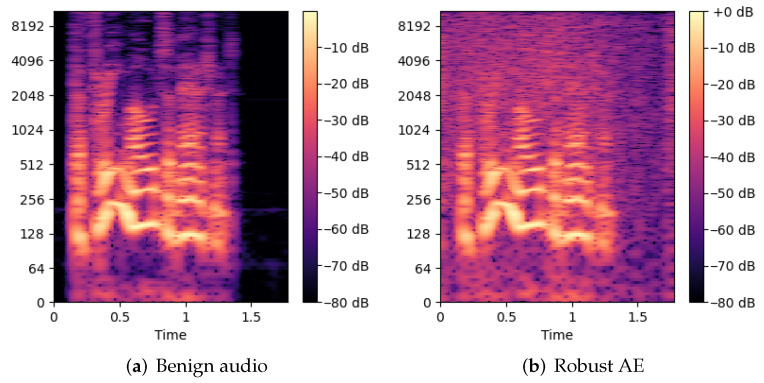
Comparing spectrograms of (**a**) benign audio with (**b**) the robust audio AE. Distortion in the AE is obvious.

In light of this, we conducted experiments to verify the existence of AEs that were robust against our method. In these experiments, we generated a robust audio AE against DeepSpeech2 in a similar way to generating our targeted audio AEs, as discussed in Section 4.1. During each epoch, we modified the audio along the gradient of the loss function’s direction, denoted as $\bar{g}$, as well as a perpendicular direction, denoted as $\bar{p}$, and optimized the audio together with the modified ones. It should be noted that $\bar{p}$ and $\bar{g}$ are also used for generating heat maps and extracting features as discussed in Section 3. Theoretically, this strategy of generating robust audio AEs is equivalent to the EoT. There are two stages in the process. Stage 1 succeeds if the robust audio AE is transcribed as the target phrase and the maximum edit distance between transcripts of all modified audio and the targeted phrase is less than 3. Then, stage 2 focuses on lowering the noise in the robust audio AE. Similar to the other experiments, we ran stage 2 for 300 epochs. We successfully generated one targeted audio AE in 23.1 min, and the corresponding heat maps are shown in Figure 5. As expected, the heat maps of this robust audio AE are similar to the benign audios shown in Figure 3 because there is only a small change in loss values and normalized edit distance when it is slightly modified along $\bar{p}$ and $\bar{g}$. Although this implies the success of this robust AE, its distortion is −11.2 dB, which is noisier than the −20 dB distortion set. To demonstrate this, Figure 6 uses spectrograms to show that the distortion is obvious.

**Figure 7:**
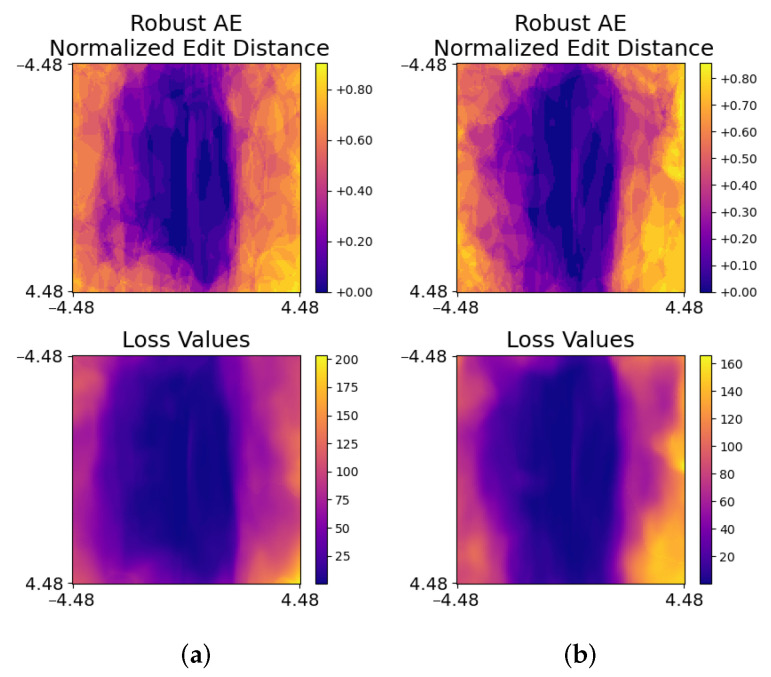
Using different values of $\bar{p}$ to generate heat maps of loss function values and normalized edit distances for a robust audio AE. Different values of $\bar{p}$ were used to generate (**a**,**b**).

Looking deeper, $\bar{p}$ is initialized from a fixed vector for the purpose of reproducibility during implementation and this is fine under a black-box threat model in which adversaries have no knowledge about $\bar{p}$. However, this fixed initialization might be exploited under a white-box threat model. Specifically, $\bar{p}$ would be almost fixed when $\bar{g}$ is stable and a robust audio AE only needs to cause a small change in loss values and normalized edit distance along the almost fixed $\bar{p}$ and $\bar{g}$. This can happen when the loss is near a local minimum or when optimization is stuck on a plateau. Hence, it is interesting to investigate whether a defender can detect the robust AE by deliberately using different values of $\bar{p}$. Figure 7 presents heat maps of the robust AE with different values of $\bar{p}$. It shows that heat maps of the robust AE still resemble heat maps of benign audio, which implies that the robust AE can bypass detection even if different values of $\bar{p}$ are used.

### 6.2. Eliminating Adversarial Examples

Research has demonstrated that the detection of adversarial perturbations in input is efficient and can achieve high performance. This makes it suitable for potential deployment in real-world applications. Nevertheless, existing detection methods cannot theoretically guarantee the perfect detection of future AEs. This means there is still a likelihood, no matter how small, that an AE can bypass detection and cause harm. Other work on defending against AEs has focused on adversarial training [62,63] and theoretical robustness [64]. However, these research directions have their own unsolved problems: the performance of adversarially trained models is unsatisfactory because of the tradeoff between performance and robustness [50], and methods that provide theoretical robustness against AEs cannot work for real-world complex DNNs. We contend that the ultimate solution for defending against AEs is to make the decision strategy of DNNs align with human perception. In this manner, AEs can be eliminated because human perception is robust against small perturbations.

There is currently a significant difference between the decision strategy of DNNs and the perception of humans. This is demonstrated by the “Clever Hans” behavior in DNN predictions [65]. Specifically, DNN predictions can be based on non-robust features that allow distinction within the training data but are not related to the intended task [19]. An example by Lapuschkin et al. [65] showed that an image classification model outputs a “horse” label if a source tag is present because one-fifth of the horse images contained this source tag. To demonstrate this, the researchers stamped the source tag onto an image of a car and the prediction changed from “car” to “horse”.

The “Clever Hans” behavior also exists in ASR systems because of the existence of audio AEs. Recall that small or even imperceptible perturbations can force an ASR system to output a malicious command that was predefined by an adversary at a 100% success rate as shown in Table 3. Geirhos et al. [66] explained this “Clever Hans” behavior in DNNs via the concept of shortcut learning. In fact, shortcut learning also exists in biological neural networks. They provided the example that rats distinguished between colors through the odor of paint, which was an unintended solution in the experiments.

In the literature, the direction of aligning the decision strategy of DNNs with human perception has attracted more and more attention from researchers in recent years. For example, Liu et al. [67] proposed first identifying incorrect predictions and then increasing their weight for retraining a DNN. Singla et al. [68] recently proposed a novel method to identify spurious features that are learned by DNNs. These examples highlight that aligning the decision strategy of DNNs with human perception for improving the robustness of models against AEs is an interesting direction for future work.

## 7. Conclusions and Future Work

With ASR systems becoming ubiquitous in commonly used commercial applications, audio AEs pose a severe security threat to these systems. Despite the various methods proposed by the research community for defending against audio AEs, the intrinsic properties of audio AEs have not been well studied or understood. This paper presents a method for visualizing the different decision boundary patterns around audio AEs and benign audio, which allow them to be distinguished from each other. This paper also showed that by extracting features based on the decision boundaries in conjunction with dimensionality-reduction techniques, the features of audio AEs and benign audio can be clearly separated in 2D space. In addition, this work demonstrated the possibility of detecting previously unknown audio AEs using anomaly detection. Our experimental results showed that this approach achieved significantly high detection rates for targeted audio AEs.

In future work, we will investigate various methods for improving audio AE detection results through the incorporation of more advanced feature-extraction techniques and anomaly-detection models. Another interesting direction for future work is on improving the robustness of models against AEs via aligning the decision strategy of DNNs with human perception.

## Figures and Tables

**Table 2:** Techniques for defending against audio AEs in ASR systems.

Defense Type	Method	Target Model	Technique
Detection	Zeng et al. [27]	DeepSpeech	Multiple transcripts
	Yang et al. [28]	DeepSpeech, Kaldi	Temporal dependency
	Samizade et al. [44]	DeepSpeech	CNN
	Guo et al. [45]	DeepSpeech	Multivariant partition
	Hussain et al. [46]	DeepSpeech, Lingvo	Input transformation
Recovery	Yang et al. [47]	DeepSpeech	Speech quality enhancement
	Guo et al. [48]	DeepSpeech	Noise reduction
	Yuan et al. [32]	Kaldi	Downsampling
	Chen et al. [40]	IBM Speech to Text	Downsampling

**Table 3:** Total time taken for generating the audio AEs and their success rates.

Type	DeepSpeech	DeepSpeech2
Targeted AEs	17.4 h (100.00%)	6.0 h (100.00%)
Untargeted AEs	11.0 h (98.68%)	12.3 h (100.00%)
FGSM AEs	0.13 h (28.79%)	0.07 h (38.66%)

**Table 4:** Anomaly detection results of previously unknown audio AEs.

	DeepSpeech					DeepSpeech2				
**Type**	**TP**	**FP**	**TN**	**FN**	**DR**	**TP**	**FP**	**TN**	**FN**	**DR**
Targeted AEs	150	-	-	0	100.00%	150	-	-	0	100.00%
Untargeted AEs	120	-	-	30	80.00%	129	-	-	21	86.00%
FGSM AEs	86	-	-	64	57.33%	33	-	-	117	22.00%
Noisy Audio	-	9	141	-	94.00%	-	8	142	-	94.67%
Correctly trans.	-	4	146	-	97.33%	-	2	148	-	98.67%
Incorrectly trans.	-	6	144	-	96.00%	-	12	138	-	92.00%
	**Pre**	**Rec**	**Acc**			**Pre**	**Rec**	**Acc**		
	94.93%	79.11%	87.44%			93.41%	69.33%	82.22%		

## Data Availability

Data from [9] is available at https://nicholas.carlini.com/code/audio_adversarial_examples (accessed on 1 November 2020); Data from [36] is available at http://cseweb.ucsd.edu/~yaq007/imperceptible-robust-adv.html (accessed on 1 November 2020); Examples from this paper are available at https://drive.google.com/drive/folders/1Ffed7xHmP5oKCuypEgJxQ80p35-vSIBm?usp=sharing (accessed on 20 October 2022).
